# Supplementary material for: The limiting zero dynamics of discrete-time system based on forward triangle sample-and-hold
Source: PLoS One. 2023 Apr 11;18(4):e0283072. doi: 10.1371/journal.pone.0283072 (PMC10089353; doi:10.1371/journal.pone.0283072)
Supplement: Attachment — Submitted filename: 34561Comments.docx [file pone.0283072.s001.docx]

**Report on "The limiting zero dynamics of discrete-time system based on forward triangle sample-and-hold"**

This study concerns with the properties of limiting zero dynamics of the resulting discrete-time systems. The results of the limiting zero dynamics in the situation of sufficiently small or large sampled period is introduced. Then, the stable conditions of the limiting zeros is presented.

The topic of this paper is interesting. The methods of this paper is novel. The organizations of this paper and the written style look well. However, a number of points need to be clarified and certain statements require further justification. There are given as below.

**Comments and remarks:**

1. **The setting of the figures is not standard. For example, the size and font of the text in the figure, the thickness of the lines, etc.**
2. I would encourage the author to extend the abstract more with the key results, and the description of highlights and main conclusions should also be improved at the end.
3. On page 14, line 359, the writing is not standardized, “As shown in Fig.??”.
4. The format of the references is not uniform, e.g. 24.

In conclusion, if the authors could revise the manuscript in the light of the mentioned above, I would like to recommend the paper for publication in the journal:" PLOS ONE".
